# Short Pretreatment with Calcitriol Is Far Superior to Continuous Treatment in Stimulating Proliferation and Osteogenic Differentiation of Human Adipose Stem Cells 

**DOI:** 10.22074/cellj.2020.6773

**Published:** 2019-12-15

**Authors:** Fatemeh Mokhtari-Jafari, Ghassem Amoabediny, Mohammad Mehdi Dehghan, Marco N. Helder, Behrouz Zandieh-Doulabi, Jenneke Klein-Nulend

**Affiliations:** 1. School of Chemical Engineering, College of Engineering, University of Tehran, Tehran, Iran; 2. Department of Biomedical Engineering, Research Center for New Technologies in Life Science Engineering, University of Tehran, Tehran, Iran; 3. Amsterdam UMC-location VUMC and Academic Centre for Dentistry Amsterdam (ACTA), Vrije Universiteit Amsterdam, Department of Oral and Maxillofacial Surgery/Oral Pathology, Amsterdam Movement Sciences, Amsterdam, The Netherlands; 4. Department of Surgery and Radiology, Faculty of Veterinary Medicine, University of Tehran, Tehran, Iran; 5. Institute of Biomedical Research, University of Tehran, Tehran, Iran; 6. Department of Oral Cell Biology, Academic Centre for Dentistry Amsterdam (ACTA), University of Amsterdam and Vrije Universiteit Amsterdam, Amsterdam Movement Sciences, Amsterdam, The Netherlands

**Keywords:** 1, 25-dihydroxy Vitamin D_3_, Adipose-derived Stem Cells, Bone, Osteogenesis, Proliferation

## Abstract

**Objective:**

This study investigated whether short stimulation (30 minutes) of human adipose stem cells (hASCs) with 1,25-dihydroxyvitamin D3(calcitriol or 1,25-(OH)_2_VitD_3_), fitting within the surgical procedure time frame, suffices to induce osteogenic differentiation, and compared this with continuous treatment with 1,25-(OH)_2_VitD_3_.

**Materials and Methods:**

In this experimental study, hASCs were pretreated with/without 10 nM calcitriol for 30 minutes, seeded on biphasic calcium phosphate (BCP), and cultured for 3 weeks with/without 1,25-(OH)_2_VitD_3_. Cell attachment was determined 30 minutes after cell seeding. AlamarBlue assay, alkaline phosphatase (ALP) assay, ALP staining, real-time polymerase chain reaction (PCR), and protein assay were used to evaluate the effect of short calcitriol pretreatment on proliferation and osteogenic differentiation of hASCs up to 3 weeks.

**Results:**

Pretreatment with 1,25-(OH)_2_VitD_3_enhanced the attachment of hASCs to BCP by 1.5-fold compared to non- treated cells and increased the proliferation by 3.5-fold at day 14, and 2.6-fold at day 21. In contrast, continuous treatment increased the proliferation by 1.7-fold only at day 14. After 2 weeks, ALP activity was increased by 18.5-fold when hASCs were pretreated with 1,25-(OH)_2_VitD_3_for 30 minutes but increased only 2.6-fold when compared with its continuous counterpart. Moreover, after 14 days, pretreatment resulted in significant upregulation of the osteogenic markers *RUNX2* and *SPARC* by 3.6-fold and 2.2-fold, respectively, while this was not observed upon continuous treatment. Finally, 30 minutes pretreatment of hASCs with 1,25-(OH)_2_VitD_3_increased *VEGF_189_* expression, which may contribute to the process of angiogenesis.

**Conclusion:**

This study is the first research showing that 30 minutes pretreatment of hASCs with 1,25-(OH)_2_VitD_3_, not only enhanced cell attachment to the scaffold at seeding time, but also promoted the proliferation and osteogenic differentiation of hASCs more strongly than continuous treatment, suggesting that short pre-treatment with 1,25-(OH)_2_VitD_3_is a promising approach for the regeneration of bones in a one-step surgical procedure.

## Introduction

Bone regeneration is a process required for various bone diseases, including degenerative diseases, orthopedic surgeries, osteonecrosis, or non-union fractures, in which reconstruction of injured bone is needed (1). Engineered bone tissue is considered a potential alternative to the customary use of bone grafts due to the boundless supply and lack of disease transmission (2). Engineering the functional bone using a combination of (stem) cells, scaffolds, and osteostimulative factors is a promising strategy for the future development of bone regeneration.

Human adipose stem cells (hASCs) are the favoured cell source for the rehabilitation of massive bone defects due to its potential to trigger osteogenic and angiogenic differentiation (3, 4). Adipose tissue can be harvested with the least discomfort to patients and easily upscaled as needed. Moreover, it contains a high number of stem cell in comparison with its volume, which allows obtaining highly enriched ASC [residing in the stromal vascular fraction (SVF)] within a short time frame. Taken together, this implies that clinically relevant stem cell quantities can be achieved instantly after adipose tissue processing in a one-step surgical procedure (5). This novel concept is not only cost-effective but also beneficial to the patients, mainly because a second surgical intervention can be avoided. Moreover, clinical results showed the efficiency, feasibility, and safety of applying autologous ASCs in the human maxillary sinus floor elevation, and high angiogenic power of SVF. The potential of ASCs to stimulate osteogenesis and angiogenesis offers a promising solution for the field of bone tissue engineering (3).

Previously, we found that within the short time frame of the one-step surgical procedure, *ex vivo* exposure to a physiological concentration (10 ng/ml) of recombinant human bone morphogenetic protein-2 (rhBMP2) for 10- 30 minutes caused a pronounced increase in proliferation and acceleration of osteogenic differentiation (6). However, rhBMP2 is rather expensive and associated with some adverse effects when not properly used (7). Since 1,25-(OH)_2_VitD_3_ is a well-known accelerator of osteoblast differentiation and mineralization (8, 9) as well as a potent osteogenic inducer of ASC differentiation and mechanoresponsiveness (10), we tested whether 1,25-(OH)_2_VitD_3_ could be a cheaper while equally effective alternative to rhBMP2.

Since 1,25-(OH)_2_VitD_3_ plays an active role in bone regeneration, many studies have investigated the effect of different types of calcitriol administration on osteogenic differentiation and bone formation. For instance, the intraperitoneal administration of 1,25-(OH)_2_VitD_3_ after implantation of beta-tricalcium phosphate (β-TCP) loaded with ASCs contributed to the increase of bone volume (11). Similarly, local administration of 1,25-(OH)_2_VitD_3_ into rat mandibular bone defects revealed significantly higher bone volume after 1 and 2 weeks and more mineralized bone and uniform collagen structure after 4 and 8 weeks (12). Although, the osteogenic markers, including alkaline phosphatase, osteopontin and osteocalcin were enhanced by 25-hydroxyvitamin D_3_ and 1,25-(OH)_2_VitD_3_ in a dose-dependent manner. Also, 10 nM 1,25-(OH)2D3 promoted ALP activity and osteogenic differentiation more than 0.05, 0.1, and 1 nM (8).

Earlier, we found that biphasic calcium phosphate scaffolds (BCP) can be used as bone substitute material for dental and orthopedic applications (13), and clinical results have shown that BCP, containing 20% HA and 80% β-TCP (BCP20/80) (Institut Straumann AG, Switzerland), might give a superior performance as a scaffold for bone augmentation in maxillary sinus floor elevation compared to BCP that composed of 40% HA and 60% β-TCP (BCP40/60), owing to more bone formation and osteoid deposition (13).

Therefore, the aim of this study was to indicate the osteogenic and angiogenic response of hASCs to short (30 minutes) pre-treatment with 1,25-(OH)_2_VitD_3_, to reveal whether this approach could promote bone regeneration. Moreover, we compared the potency of 1,25-(OH)_2_VitD_3_ for osteogenic induction in this short-term stimulation protocol, to continuous stimulation with the factor.

## Materials and Methods

### Biphasic calcium phosphate scaffolds

In this experimental study, Straumann Bone Ceramic 20/80 (Institut Straumann AG, Switzerland), a custommade porous BCP scaffold that composed of 20% HA and 80% β-TCP (BCP20/80) was used as a scaffold. The particle properties include the size range between 500 and 1000 µm, micro-porosity 2%, interconnected pores between 100 and 500 µm, and porosity 90%. The crystal size of BCP 20/80 was 1.0-6.0 µm, and the granules had a specific surface area of 9.5×10^-3^ m^2^/g. Surface morphology and characteristics have been previously reported (14).

### Donors

Subcutaneous adipose tissue was obtained from residues of abdominal wall resections belonging to 3 healthy female donors (age: 33, 40, 47), who underwent elective surgery for abdominal wall correction at the Tergooi Hospital Hilversum and a clinic in Bilthoven, The Netherlands. The Ethical Review Board of the Vrije Universiteit (VU) Amsterdam University Medical Center, The Netherlands, confirmed the study protocols. All patients signed informed consent. Phenotypical and functional characterizations of freshly isolated adipose tissue-derived stem cells have been reported previously by our group (11).

### 1,25-(OH)_2_VitD_3_ treatment and human adipose stem cells attachment to biphasic calcium phosphate scaffolds

The isolation of hASCs has been described earlier (6). Pooled hASCs from 3 donors at passage 3 were used. hASCs were either or not incubated with 10^-8^ M 1,25-(OH)_2_VitD_3_ at room temperature for 30 minutes. Then, the cells were washed twice with PBS to remove 1,25-(OH)_2_VitD_3_, centrifuged, and resuspended in Dulbecco’s Minimum Essential Medium (DMEM, Gibco, Life Technologies, USA) without any supplements. Cells were seeded at the density of 5.5×10^4^ cells per 25-35 mg of BCP20/80 scaffold in 2 mL tubes (Eppendorf Biopur®, Germany), and allowed to adhere for 30 minutes to the scaffolds. After washing twice with PBS, scaffolds with attached cells were transferred into 12-well plates with Costar® Transwell® containers (Corning Life Sciences, Lowell, MA, USA) containing expansion medium (DMEM) supplemented with 10% fetal clone I (FCI, ThermoFisher Scientific, USA) as an alternative to fetal bovine serum (FBS), antibiotics [1% penicillin/streptomycin/fungizone (PSF)), 50 μM ascorbic acid (Merck, Germany), and 10 mM β-glycerol phosphate (Merck, Germany). The hASCs-seeded scaffolds were incubated at 5% CO_2_ in a humidified incubator at 37˚C for 3 weeks.

### DNA quantification

hASCs were treated for 30 minutes with 10^-8^ M 1,25-(OH)_2_VitD_3_, seeded on BCP20/80, and following the initial attachment for 30 minutes, BCP20/80 was washed with PBS, and the number of detached cells was measured. Unattached hASC from the washing steps were centrifuged and lysed in cOmplete™ Lysis-M buffer (Roche Laboratories, IN, USA) for DNA quantification using the Cyquant Cell Proliferation Assay Kit (Molecular Probes/Invitrogen, Carlsbad, CA, USA) according to the manufacturer’s protocols. Absorption was read at 480 nm excitation and 520 nm emission in a Synergy HT spectrophotometer (BioTek Laboratories, PA, USA).

### Human adipose stem cell proliferation on biphasic calcium phosphate scaffolds

Proliferation was assessed using AlamarBlue® fluorescent assay (Invitrogen, Frederick, MD, USA), at day 4, 14, and 21, according to the manufacturer’s instructions. We observed a linear relationship between AlamarBlue fluorescence and the cell number (data not shown). Fluorescence was measured in medium samples at 530 nm excitation and 590 nm emission using a Synergy HT spectrophotometer.

### Colony-forming unit assay

Colony-forming unit assay (CFU) was performed to assess the colony forming capacity of hASCs in hASC culture at passage 3. Cells were seeded in 6-well plates (Greiner Bio-One^TM^, Alphen a/d Rijn, The Netherlands) at concentrations of 1, 5, 10, 50, and 100 cells/well. After 14 days of culture, 4% formaldehyde was prepared to fix the cells, and then 0.2% toluidine blue in the borax buffer (PH=12) was used for 1 minute to stain the cells. A colony was specified as a visible mass of the cells which composed of more than 10 clustered cells. Colony counting was performed under a light microscope at 100x magnifications. The percentage of CFU per total number of hASCs was reported.

### Alkaline phosphatase activity

Alkaline phosphatase (ALP) activity can signify the initiation of osteogenic differentiation of hASC seeded on BCP20/80 scaffolds. After 4, 14, and 21 days of culture, scaffolds were transferred into 24-well culture plates (Cellstar, Germany) and washed with PBS. The cells were lysed with cOmplete™ Lysis-M buffer to assess ALP activity and protein contents. P-nitrophenylphosphate (Fluka, Poole, UK) at pH=10.3 was designated as the substrate for ALP. The absorbance was read at 405 nm. ALP activity was normalized to cellular protein and expressed as µmoles of p-nitrophenol formed per hour per milligram of cellular protein. After 4, 7, and 14 days of culture, ALP activity was also visualized using nitro blue tetrazolium chloride/5-bromo-4-chloro-3-indolyl phosphate (NBT/BCIP; Roche, Germany) following the standard protocols. Assessment of protein content was carried out by a BCA Protein Assay Reagent Kit (PierceTM, Rockford, III, USA), and the absorbance was measured at 540 nm with a Synergy HT spectrophotometer.

### Analysis of gene expression

Total RNA was isolated from hASCs (from 3 donors) cultured on BCP20/80 scaffolds for 4, 14, and 21 days, using TRIzol® reagent (Life Technologies^TM^) according to the manufacturer’s instructions, and stored at -80˚C until further use. cDNA was synthesized using a thermocycler GeneAmp® PCR System9700 PE (Applied Biosystems, Foster City, CA, USA), using SuperScript® VILO^TM^ cDNA Synthesis Kit (Life Technologies^TM^, USA) with 0.1 µg total RNA in a 20 µL reaction mix containing VILOTM Reaction Mix and SuperScript® Enzyme Mix. cDNA was stored at -20˚C before the real-time PCR analysis.

Real-time PCR reactions were run in a LightCycler® (Roche Diagnostics) using 1 µL of 5x diluted cDNA and SYBR® Green Mastermix (Roche Laboratories, IN, USA), according to the manufacturer’s protocols, for the following cycles: 10 minutes pre-incubation at 95˚C, followed by 45 cycles of amplification at 95˚C for 2 seconds, 56˚C for 8 seconds, 72˚C for 10 seconds, and 82˚C for 5 seconds, after which melting curve analysis was performed. In each run, the reaction mixture without cDNA was used as the negative control. All primers used for realtime PCR were procured from Life Technologies^TM^ (Table 1). The relative gene expression was normalized against the relative human 14-3-3 protein zeta/delta (*YWHAZ*) and hypoxanthine-guanine phosphoribosyltransferase (*HPRT*) as housekeeping genes. Real-time polymerase chain reaction (PCR) was used to determine the expression of Runt-related transcription factor 2 (*RUNX2*), ALP, osteonectin (*SPARC*), osteopontin (*OPN*), dentin matrix acidic phosphoprotein 1 (*DMP1*), proliferation marker ki-67, vitamin D nuclear receptor VDR, cytochrome p450-enzyme (*CYP24*), and vascular endothelial growth factor (*VEGF*). In each assay, for osteogenic markers, cDNA from osteoblasts or human reference (Agilent Technologies, Stratagene Products Division, La Jolla, CA, USA) was used as the reference DNA. Crossing points were plotted versus the serial dilutions of the known concentrations of the reference DNA (2.5-0.004 ng/μL) using the Light Cycler^®^ software (version 1.2). The gene expression analysis was studied between the cells treated with or without 1,25-(OH)_2_VitD_3_ treatment.

### Statistical analysis

The obtained data were analyzed by the GraphPad software version 5 (GraphPad Software, USA) and expressed as the means and standard error of the mean (mean ± SEM). To assess the statistical significance between the experimental groups, Student’s t test, and two-way analysis of variance (ANOVA) were conducted where appropriate. The level of significance was set at P<0.05. All experiments were performed in triplicate.

## Results

### Human adipose stem cell attachment to biphasic calcium phosphate scaffold

**Fig 1:**
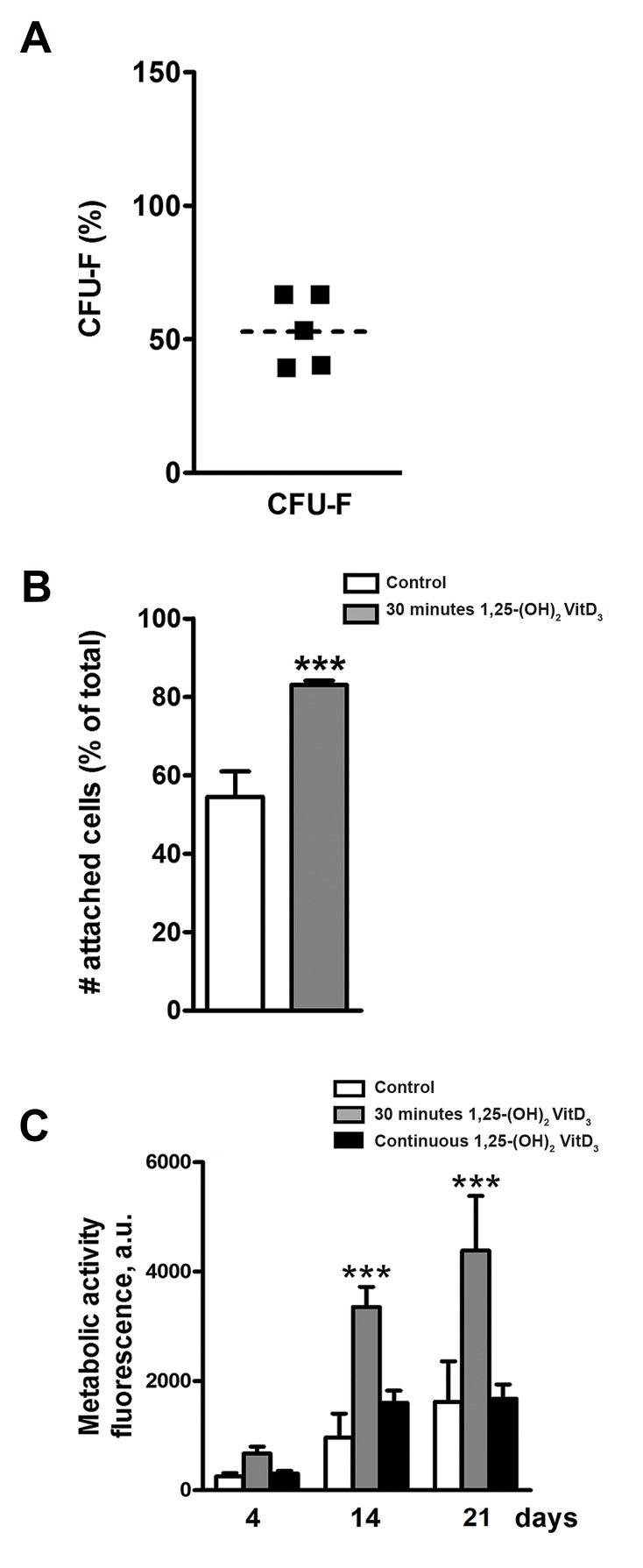
hASC attachment to BCP20/80 scaffold with or without 1,25-(OH)_2_VitD_3_ at day 0 and the effect of 1,25-(OH)_2_VitD_3_ treatment on the metabolic activity of hASCs. **A.** The average of CFU of non-treated hASCs cultured on tissue culture plastic for 2 weeks was nearly 53% (dotted line), **B.** Cell attachment to BCP after 30 minutes pre-treatment with 10-8 M 1,25-(OH)_2_VitD_3_ was significantly increased compared to controls, and C. Thirty minutes incubation with 1,25-(OH)_2_VitD_3_ significantly increased the proliferation after 14 and 21 days compared to continuous treatment with 1,25-(OH)_2_VitD_3_. Values are expressed as mean ± SEM (n=3). hASCs; Human adipose stem cells, BCP; Biphasic calcium phosphate, CFU; Colony forming unit, and ***; Significantly different from control, P<0.001.

The number of CFU was counted 14 days after the cell culture of non-treated hASCs on tissue culture plastic. CFU-f frequency of non-treated hASCs was around 53%, representing the number of viable hASCs in adipose tissue (Fig .1A). Pretreatment of hASCs with 1,25-(OH)_2_VitD_3_ for 30 minutes increased the attachment of cells to BCP20/80 scaffolds by 1.5-fold (from 54 to 83%) at seeding time compared to nontreated cells (Fig .1B).

**Table 1:** Primer sequences for the evaluation of angiogenesis and osteogenesis through real-time polymerase chain reaction


Target gene (human)	Primer sequence (5ˊ-3ˊ)

*YWHAZ*	F: GATGAAGCCATTGCTGAACTTG
	R: CTATTTGTGGGACAGCATGGA
*HPRT*	F: GCTGACCTGCTGGATTACAT
	R: CTTGCGACCTTGACCATCT
*RUNX2*	F: ATGCTTCATTCGCCTCAC
	R: ACTGCTTGCAGCCTTAAAT
*ALP*	F: AGGGACATTGACGTGATCAT
	R: CCTGGCTCGAAGAGACC
*SPARC*	F: CTGTCCAGGTGGAAGTAGG
	R: GTGGCAGGAAGAGTCGAAG
*Ki-67*	F: CCCTCAGCAAGCCTGAGAA
	R: AGAGGCGTATTAGGAGGCAAG
*OPN*	F: TTCCAAGTAAGTCCAACGAAAG
	R: GTGACCAGTTCATCAGATTCAT
*DMP1*	F: TAGGCTAGCTGGTGGCTTCT
	R: AACTCGGAGCCGTCTCCAT
*VDR*	F: GACACAGCCTGGAGCTGAT
	R: CAGGTCGGCTAGCTTCTGGA
*CYP24a1*	F: AGCCTGCTGGAAGCTCTGTACC
	R: TGTTCAGCTCGCTGTACAAGTC
*VEGF189*	F: ATCTTCAAGCCATCCTGTGTGC
	R: CACAGGGAACGCTCCAGGAC


### Effect of 30 minutes pre-treatment with calcitriol on human adipose stem cell proliferation

Thirty minutes pre-treatment with calcitriol significantly increased the cell number after 2 and 3 weeks compared to continuous treatment. Thirty minutes pre-treatment with 1,25-(OH)_2_VitD_3_ increased the cell number at day 14 by 3.5-fold, and at day 21 by 2.6-fold. Continuous treatment with 1,25-(OH)_2_VitD_3_ for 3 weeks increased the cell number only at day 14 by 1.7-fold, but not at day 21 compared to non-treated controls (Fig .1C).

### Effect of 30 minutes pre-treatment with 1,25-(OH)_2_VitD_3_ on alkaline phosphatase activity in human adipose stem cells

**Fig 2:**
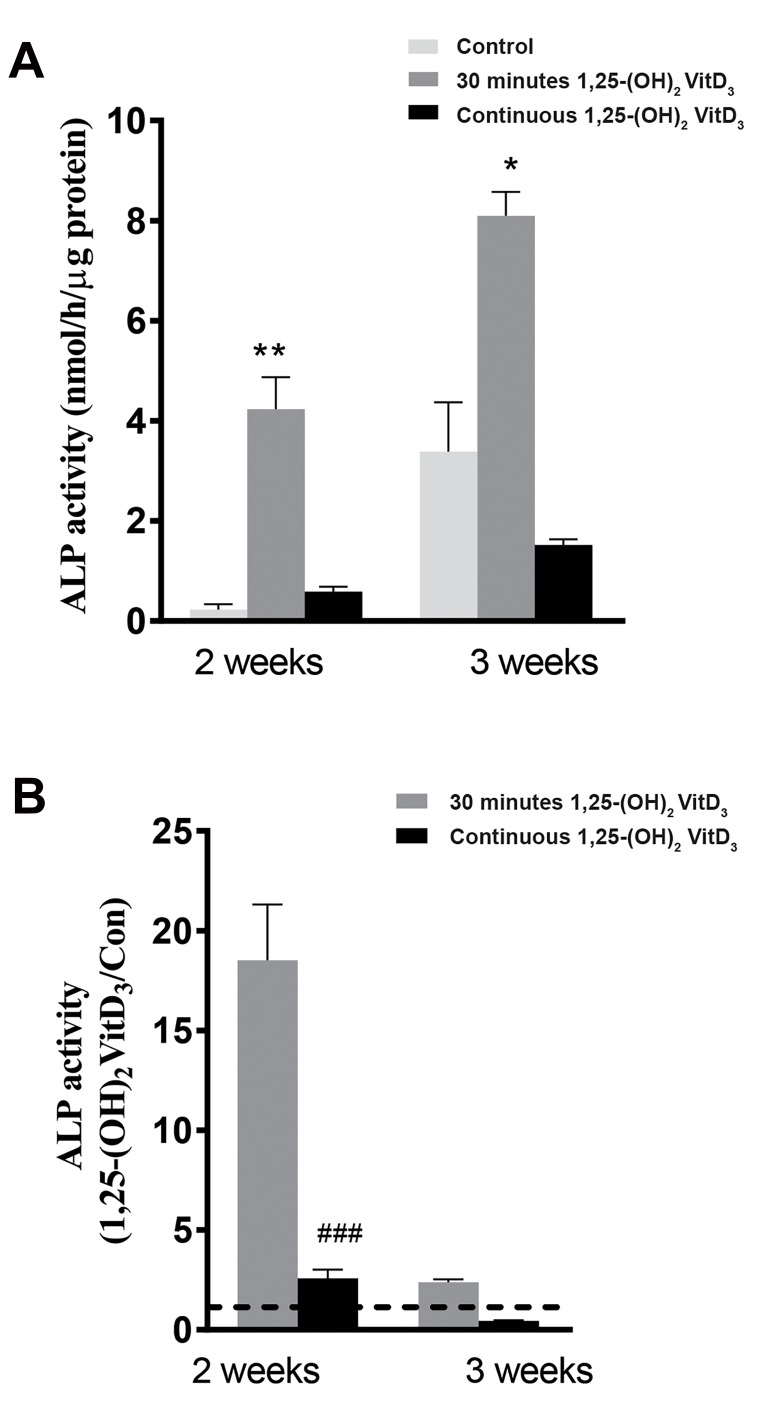
Short (30 minutes) versus long (3 weeks) 1,25-(OH)_2_VitD_3_ treatment effects on ALP activity in hASCs. Thirty minutes incubation with 1,25-(OH)_2_VitD_3_ increased ALP activity after 2 weeks (18.5-fold) and 3 weeks (2.4-fold). Continuous treatment with 10-8 M 1,25-(OH)_2_VitD_3_ increased ALP activity after 2 weeks (2.6-fold), but not at 3 weeks (0.4-fold). Values are presented as mean ± SEM (n=3). ALP; Alkaline phosphatase, hASCs; Human adipose stem cells, *; Significantly different from control, P<0.05, **; P<0.01, and ###; Significantly different from 30 minutes 1,25-(OH)_2_VitD_3_, P<0.001.

**Fig 3:**
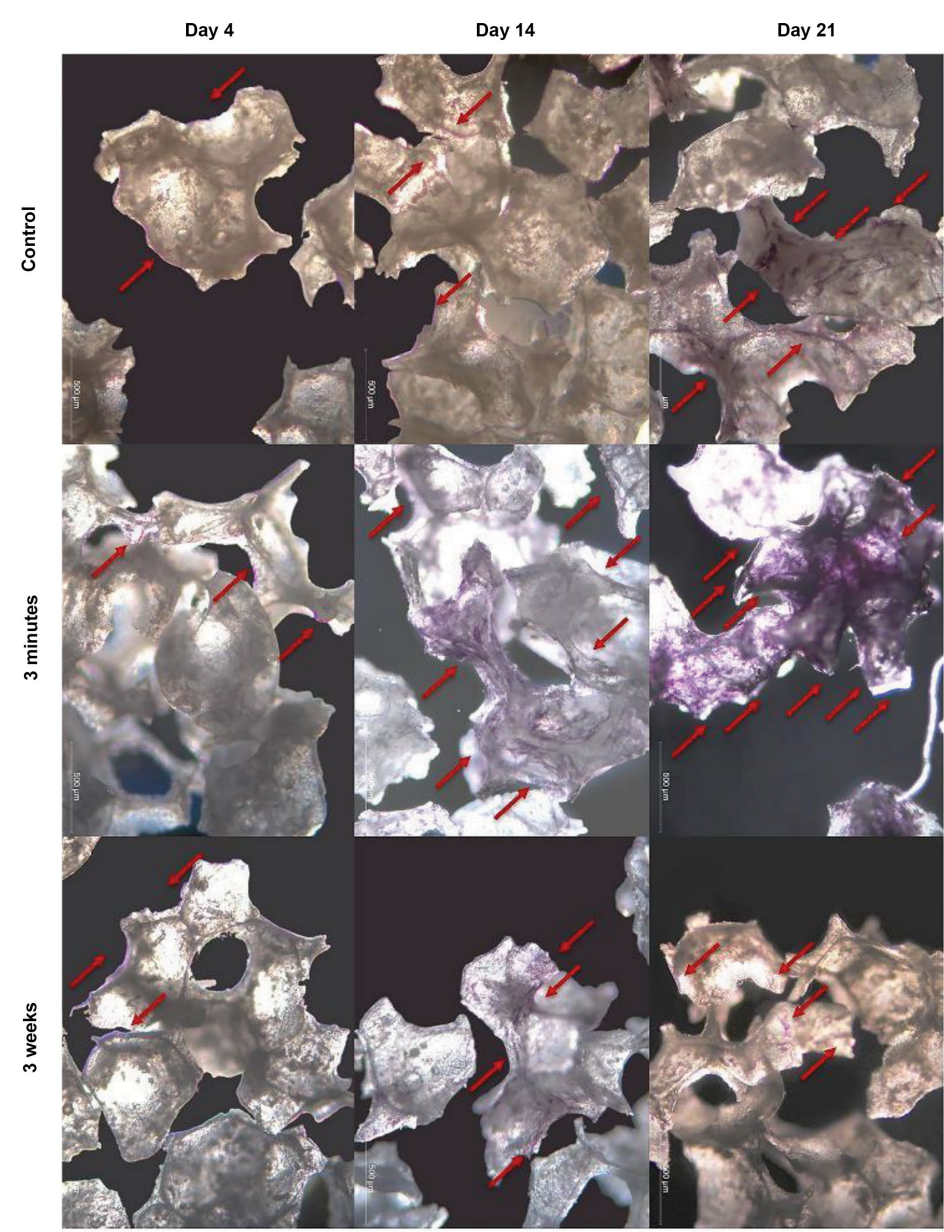
The effects of short (30 minutes) versus long (3 weeks) treatment with 1,25-(OH)_2_VitD_3_ on ALP activity. hASCs were stained to detect ALP activity using NBT/BCIP. Pretreatment with 1,25-(OH)_2_VitD_3_ for 30 minutes notably increased ALP activity after 2 and 3 weeks compared to the continuous treatment. Red arrows show ALP activity of hASCs. ALP; Alkaline phosphatase, hASCs; Human adipose stem cells, and NBT/BCIP; Nitro blue tetrazolium chloride/5-bromo- 4-chloro-3-indolyl phosphate (scale bar: 500 μm).

Thirty minutes pre-treatment of hASCs with 1,25-(OH)_2_VitD_3_ significantly increased ALP activity in hASCs after 2 and 3 weeks of the cell culture compared to continuous treatment with 1,25-(OH)_2_VitD_3_ and nontreated hASCs. ALP activity in hASCs after 30 minutes pre-treatment with 1,25-(OH)_2_VitD_3_ was increased by 18.5-fold compared to non-treated cells after 2 weeks, while ALP activity of continuous treatment with 1,25-(OH)_2_VitD_3_ was increased 2.6-fold compared to nontreated cells after 2 weeks (Fig .2). This was confirmed by ALP staining after 14 days of the cells culture (Fig .3).

### Effect of 30 minutes pre-treatment with 1,25-(OH)_2_VitD_3_ on osteogenic gene expression in human adipose stem cells

**Fig 4:**
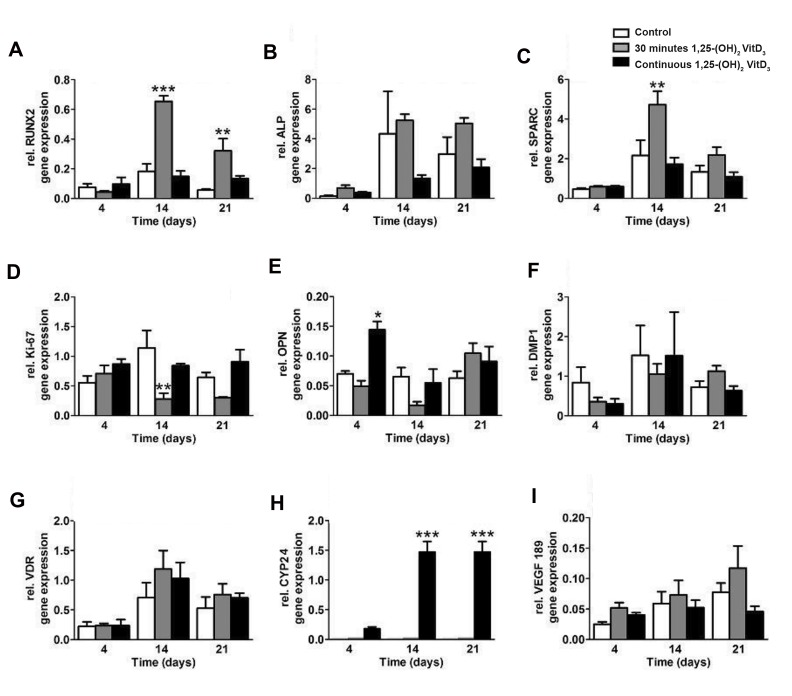
The impact of short (30 minutes) versus long (3 weeks) treatment of hASCs with 1,25-(OH)_2_VitD_3_ on osteogenic gene expression in hASCs. **A.** 30 minutes incubation with 1,25-(OH)_2_VitD_3_ increased *RUNX2* (early osteogenic marker), **B.** *ALP* (intermediate osteogenic marker) expression in hASCs after 2 and 3 weeks compared to continuous treatment with 1,25-(OH)_2_VitD_3_, **C.** 30 minutes pretreatment with 1,25-(OH)_2_VitD_3_ upregulated *SPARC* (late osteogenic marker) expression at day 14. The treatment with 1,25-(OH)_2_VitD_3_ also affected the expression of **D.** *ki-67* (proliferative marker), **E. **OPN (intermediate osteogenic marker), **F.** *DMP1* (late osteogenic marker), **G.** *VDR*, **H.** *CYP24*, and **I. ***VEGF_189_* in hASCs. Values are expressed as mean ± SEM (n=3). ALP; Alkaline phosphatase, hASCs; Human adipose stem cells, BCP; Biphasic calcium phosphate, *; Significantly different from control, P<0.05, **; P<0.01, and ***; P<0.001.

The stimulatory effect of 30 minutes pretreatment with 1,25-(OH)_2_VitD_3_ on osteogenic gene expression in hASCs seeded on BCP20/80 at day 21 was more pronounced than that of continuous treatment with 1,25-(OH)_2_VitD_3_. The expression of the *Runx2* gene, which is well-known as master transcriptional regulator of skeletogenesis (15), was analyzed and found that thirty minutes pretreatment of hASCs with 1,25-(OH)_2_VitD_3_ increased the expression of this gene (early osteogenic marker) by 3.6-fold after 2 weeks, and 5.7-fold after 3 weeks compared to non-treated hASCs. However, continuous treatment with 1,25-(OH)_2_VitD_3_ decreased *RUNX2* expression by 0.81-fold after 2 weeks and increased 2.4-fold after 3 weeks (Fig .4A). Thirty minutes pretreatment with 1,25-(OH)_2_VitD_3_ upregulated *ALP* expression, as an early marker of osteoblastic differentiation, in hASCs seeded on BCP20/80 (Fig .4B). Thirty minutes pre-treatment with 1,25-(OH)_2_VitD_3_ increased *SPARC* expression by 2.1-fold at day 14, while continuous treatment decreased the expression of *SPARC* by 0.8-fold (Fig .4C). *SPARC* regulates the activity of osteoblasts and osteoclasts, and it is expressed in osteoblasts undergoing active matrix deposition (16).

The expression of a proliferation marker *Ki-67* was decreased in cells pre-treated with 1,25-(OH)_2_VitD_3_ for 30 minutes but did not change when treated with 1,25-(OH)_2_VitD_3_ in continuous mode during 3 weeks of the cell culture (Fig .4D). The gene expression of *OPN*, which is considered crucial for bone remodeling and bio-mineralization (17), was upregulated in continuous treatment at day 4, whereas 30 minutes pretreatment of hASCs with 1,25-(OH)_2_VitD_3_ increased *OPN* expression at day 21 (Fig .4E). A gradual; however, no significant increase in *DMP1* gene expression, was observed over time (Fig .4F). *DMP1* is a highly-expressed bone extracellular matrix protein that regulates both bone development and phosphate metabolism (18).

1,25-(OH)_2_D_3_ exerts its actions via a nuclear vitamin D receptor (*VDR*), and it is regarded as the most active form of vitamin D (8). Thirty minutes pre-treatment with 1,25-(OH)_2_VitD_3_ increased *VDR* gene expression in hASCs compared to continuous treatment, with maximal stimulation at day 14 (Fig .4G). Continuous treatment with 1,25-(OH)_2_VitD_3_ increased, interestingly, the *CYP24* gene, associated with inactivation of vitamin D_3_. *CYP24*, as one of the most vitamin D-responsive genes (8), was not expressed in non-treated controls and cells pretreated with 1,25-(OH)_2_VitD_3_ for 30 minutes, but significantly increased in cells treated with 1,25-(OH)_2_VitD_3_ in a continuous mode for 3 weeks (Fig .4H). The expression of the *VEGF_189_* gene was increased in hASCs pre-treated cells with 1,25-(OH)_2_VitD_3_ for 30 minutes but reached almost at baseline in cells in a continuous treatment mode. The expression of *VEGF_189_* in cells pretreated with 1,25-(OH)_2_VitD_3_ for 30 minutes was increased by 1.5- fold at day 21, but decreased in the continuous treatment method by 0.6-fold compared to non-treated hASCs (Fig .4I).

## Discussion

In the current study, we evaluated whether a short pre-treatment of hASCs with 1,25-(OH)_2_VitD_3_ would result in a prolonged stimulatory effect on osteogenic differentiation *in vitro*. The ultimate goal was to move one step closer to the one-step surgical procedure, as described earlier (13). We found that hASCs showed differential responses after pre-treatment of hASCs with 10^-8^ M 1,25-(OH)_2_VitD_3_ for 30 minutes. More specifically, we observed that i. Pre-treated hASCs with 1,25-(OH)_2_VitD_3_ adhered better to BCP20/80 scaffolds compared to nontreated hASCs, ii. Proliferation and several osteogenic differentiation markers (ALP activity, *RUNX2*, and *SPARC* gene expression) were significantly enhanced when pretreated with1,25-(OH)_2_VitD_3_ for 30 minutes compared to control treatment, iii. The effect of short (30 minutes) pre-treatment of hASCs with 1,25-(OH)_2_VitD_3_ on osteogenic differentiation was more pronounced compared to continuous treatment with 1,25-(OH)_2_VitD_3_, and (iv) 30 minutes pre-treatment with 1,25-(OH)_2_VitD_3_ may contribute to the promotion of angiogenesis.

We found the rapid attachment of hASCs to BCP scaffolds, which was in agreement with previous findings by our group for other types of scaffolds consisting of polymeric, collagenous (19), β-TCP, and BCP20/80 biomaterials (slightly a higher attachment rate compared to β-TCP) (6). Interestingly, our data indicated a significantly higher attachment rate for the pre-treated hASCs on BCP scaffolds (1.5-fold) when compared with non-treated hASCs, which is in contrast to findings by Overman and colleagues, who found no effect of bone morphogenetic protein-2 (BMP-2), a member of the transforming growth factor-b superfamily, on attachment in an identical setting (6). Hence, 30 minutes pre-treatment with 1,25-(OH)_2_VitD_3_ appears superior to BMP2 in this regard, which may benefit the one-step surgical procedure.

Calcitriol plays an autocrine or a paracrine role in the local regulation of cell proliferation and differentiation (8). The increase in cell proliferation of hASCs pre-treated with1,25-(OH)_2_VitD_3_ for 30 minutes was noticeable after 2 and 3 weeks of the incubation period. On the other hand, Three-week continuous treatment significantly decreased the proliferation rate, which is in line with the findings by others using ASCs (20) and primary rat osteoblasts (10). Therefore, enhancement of cell proliferation through 30 minutes pre-treatment with 1,25-(OH)_2_VitD_3_ seems promising for implantation in vivo due to the enhanced extracellular matrix formation and consequently, bone formation.

We found that the impact of 30 minutes pre-treatment with 1,25-(OH)_2_VitD_3_ on osteogenic differentiation and ALP activity was more pronounced after 14 days of the cell culture compared to the culture period at day 4 and 21, indicating a time-dependency of the stimulation of hASCs by 1,25-(OH)_2_VitD_3_. The results of continuous treatment with 1,25-(OH)_2_VitD_3_ have also been reported in other studies performed on MC3T3-E1 cells (18), Primary rat osteoblasts (10), mesenchymal stem cells derived from human alveolar periosteum (21), hASCs (22), human dental pulp, and dental follicle cells (23), which are in agreement with our current data. Nevertheless, our findings showed, for the first time, that following 14 days of incubation, ALP activity was significantly increased in hASCs pre-treated with 1,25-(OH)_2_VitD_3_ for 30 minutes compared to cells treated with 1,25-(OH)_2_VitD_3_ in a continuous treatment mode.

Most of the biological activities of 1,25-(OH)_2_VitD_3_, including cell proliferation and differentiation, are considered to be exerted through the *VDR*-mediated control of target genes (24). Moreover, silencing VDR caused a significant decrease in mineralized bone volume after the treatment with 1,25-(OH)_2_VitD_3_ (25). *VDR* gene expression was slightly higher in hASCs pretreated with 1,25-(OH)_2_VitD_3_ for 30 minutes, but had no significant differences when compared between the groups. However, the upregulation of CYP24 gene expression was observed in hASCs continuously treated with 1,25-(OH)_2_VitD_3_, but not in hASCs in pretreatment method as well as control cells, suggesting an alternative explanation. We speculate that the upregulation of the *CYP24* gene may have resulted in the inactivation of 1,25-(OH)_2_VitD_3_ as a consequence of the long-term treatment with 1,25-(OH)_2_VitD_3_, a mechanism that has also been reported earlier (24, 26). Also, the upregulation of *CYP24* by continuous treatment with 1,25-(OH)_2_VitD_3_ may also explain the findings in the study of De Kok et al. (21), who found that continuous treatment failed to induce bone formation in mesenchymal stem cells pretreated with 1,25-(OH)_2_VitD_3_.

Thirty minutes pre-treatment with 1,25-(OH)_2_VitD_3_ enhanced the expression of *VEGF_189_*. *VEGF_189_* stimulates the endothelial cell proliferation and migration in vitro and contributes to the promotion of angiogenesis. Interestingly, *VEGF* participates in the coupling of osteogenesis to angiogenesis and bone healing during different phases of bone repair (16). The expression of *VEGF* is correlated with osteoblastic differentiation, and it is downregulated at the initiation of osteoblastogenesis, while during mineralization, its expression reaches at the highest levels (27). Continuous treatment (21 days) adversely influenced the gene expression of *VEGF_189_* in hASCs to a level even below that of non-treated hASCs.

## Conclusion

This study demonstrated that 30 minutes stimulation with a low physiological dose of 1,25-(OH)_2_VitD_3_ (10^-8^ M) is sufficient to promote cell attachment to BCP20/80 scaffolds compared to non-treated cells. Moreover, short pre-treatment with calcitriol showed higher proliferation and osteogenic responses than other treatment protocols, including continuous treatment or non-treatment methods. Furthermore, short pre-treatment (30 minutes) with 1,25-(OH)_2_VitD_3_ is expected to promote angiogenesis in bone tissue-engineered constructs. Our findings indicate that a short pre-treatment with 1,25-(OH)_2_VitD_3_ could be a promising solution for a one-step surgical procedure. These results will be extrapolated and implemented in the future development of treatment strategies for large bone defects.
